# Supplementary material for: A revised biosynthetic pathway for the cofactor F420 in prokaryotes
Source: Nat Commun. 2019 Apr 5;10:1558. doi: 10.1038/s41467-019-09534-x (PMC6450877; doi:10.1038/s41467-019-09534-x)
Supplement: Supplementary file 1 — Supplementary Information [file 41467_2019_9534_MOESM1_ESM.pdf]

SUPPLEMENTARY INFORMATION

A revised biosynthetic pathway for the cofactor F_{420} in prokaryotes

Ghader Bashiri[1*#], James Antoney[2,3#], Ehab N. M. Jirgis[1], Mihir V. Shah[2], Blair Ney[2,3], Janine Copp[4], Stephanie M. Stuteley[1], Sreevalsan Sreebhavan[5], Brian Palmer[5], Martin Middleditch[1], Nobuhiko Tokuriki[4], Chris Greening[2,6], Colin Scott[2*], Edward N. Baker[1], Colin J. Jackson[2,3*]

[1]School of Biological Sciences and Maurice Wilkins Centre for Molecular Biodiscovery, The University of Auckland, Auckland 1010, New Zealand

[2]Synthetic Biology Future Science Platform, CSIRO Land & Water, Canberra, ACT, Australia

[3]Research School of Chemistry, Australian National University, Acton, Australian Capital Territory, Australia

[4]Michael Smith Laboratories, University of British Columbia, Vancouver, BC, V6T 1Z4, Canada.

[5]Auckland Cancer Society Research Centre, Faculty of Medical and Health Sciences, The University of Auckland, Auckland 1010, New Zealand

[6]School of Biological Sciences, Monash University, Clayton, Victoria, Australia

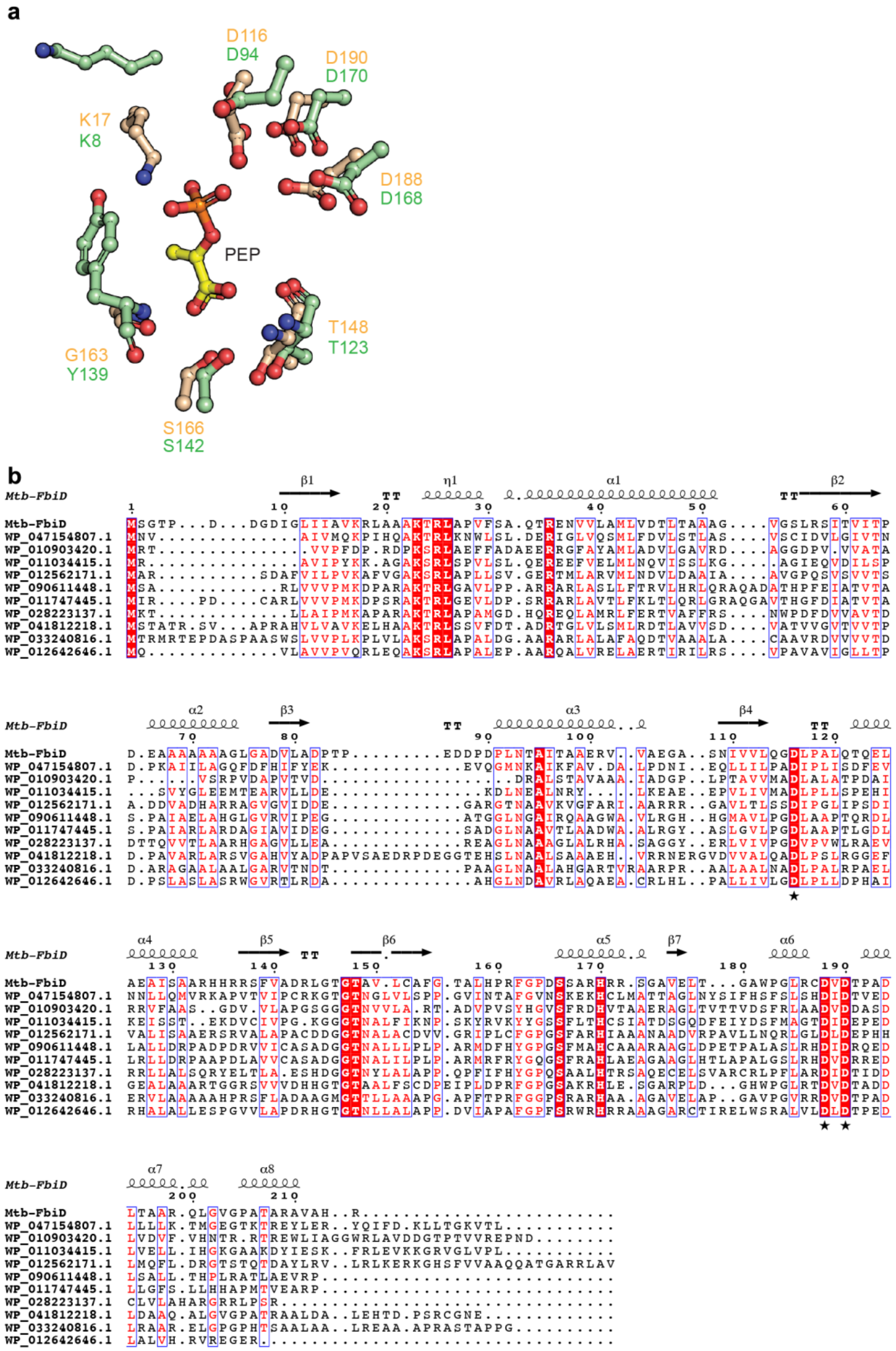

a
D116
D94
D190
D170
K17
K8
D188
D168
PEP
G163
Y139
T148
T123
S166
S142
b
Mtb-FbiD
β1 η1 α1 β2
1 10 20 30 40 50 60
Mtb-FbiD MSGTP...D...DGDIGLIIAVKRLAAAKTRLAPVFSA.QTRENVVLAMLVDTLTAAAG....VGSLRSITVITP
WP_047154807.1 MNV.............AIVMQKPIHQAKTRLKNWLSL.DERIGLVQSMLFDVLSTLAS....VSCIDVLGIVTN
WP_010903420.1 MRT..............VVPFDP.RDPKSRLAEFFADAEERRGFAYAMLADVLGAVRD....AGGDPV.VVATA
WP_011034415.1 MR...............AVIPYKK.AGAKSRLSPVLSL.QEREEFVELMLNQVISSLKG.....AGIEQVDILSP
WP_012562171.1 MAR............SDAFVILPVKAFVGAKSRLAPLLSV.GERTMLARVMLNDVLDAAIA....AVGPQSVSVVTS
WP_090611448.1 MSA.............RLVVVPMKDPARAKTRLGAVLPP.ARRARLASLLFTRVLHRLQRAQADATHPFEIATVTA
WP_011747445.1 MIR....PD....CARLVVVPMKDPSRAKTRLGEVLDP.SRRARLAVTLFKLTLQRLGRAQGAVTHGFDIATVTA
WP_028223137.1 MKT............LLAIPMKAPARAKTRLAPAMGD.HQREQLAMRLFERTVAFFRS....NWPDFDVVAVTD
WP_041812218.1 MSTATR.SV...APRAHVLVAVKELHAAKTRLSSVFDT.ADRTGLVLSMLRDTLAVVSD....VATVVGVTVVTP
WP_033240816.1 MTRMRTEPDASPAASWSLVVPLKPLVLAKSRLAPALDG.AARARLALAFAQDTVAAALA....CAAVRDVVVVTD
WP_012642646.1 MQ............VLAVVPVQRLEQAKSRLAPALEP.AARQALVRELAERTIRILRS....VPAVAVIGLLTP
α2 β3 α3 β4
70 80 90 100 110 120
Mtb-FbiD D.EAAAAAAAGLGADVLADPTP.........EDDPDPLNTAITAAERV..VAEGA..SNIVVLQGDLPALQTQEL
WP_047154807.1 D.PKAIILAGQFDFHIFYEK............EVQGMNKAIKFAV.DA.LPDNI..EQLLILPADIPLISDFEV
WP_010903420.1 P.....VSRPVDAPVTVD.................DRALSTAVAAA.IADGP..LPTAVVMADLALATPDAI
WP_011034415.1 ...SVYGLEEMTEARVLLDE..............KDLNEALNRY.....LKEAE..EPVLIVMADLPLLSPEHI
WP_012562171.1 A.DDVADHARRAGVGVIDDE............GARGTNAAVKVGFARI.AARRR..GAVLTLSSDIPGLIPSDI
WP_090611448.1 S.PAIAELAHGLGVRVIPEG............ATGGLNGAIRQAAGWA.VLRGH..HGMAVLPGDLAAPTQRDL
WP_011747445.1 S.PAIARLARDAGIAVIDEG............SADGLNAAVTLAADWA.ALRGY..ASLGVLPGDLAAPTLGDL
WP_028223137.1 DTTQVVTLAARHGAGVLLEA............REAGLNAAAGLALRHA.SAGGY..ERLVIVPGDVPVWLRAEV
WP_041812218.1 D.PAVARLARSVGAHVYADPAPVSAEDRPDEGGTEHSLNAALSAAAEH..VRRNERGVDVVALQADLPSLRGGEF
WP_033240816.1 D.ARAGAALAALGARVTNDT............PAAGLNAALAHGARTVRAARPR..AALAALNADLPALRPAEL
WP_012642646.1 D.PSLASLASRWGVRTLRDA............AHGLNDAVRLAQAEA.CRLHL..PALLIVLGDLPLLDPHAI
α4 β5 β6 α5 β7 α6
130 140 150 160 170 180 190
Mtb-FbiD AEAISAARHHRRSFVADRLGTGTAV.LCAFG.TALHPRFGPDSSARHRR.SGAVELT...GAWPGLRCDVDTPAD
WP_047154807.1 NNLLQMVRKAPVTVIPCRKGTGTNGLVLSPP.GVINTAFGVNSKEKHCLMATTAGLNYSIFHSFSLSHDIDTVED
WP_010903420.1 RRVFAAS..GDV.VLAPGSGGGTNVVLA.RT.ADVPVSYHGVSFRDHVTAAERAGLTVTTVDSFRLAADVDDASD
WP_011034415.1 KEISST..EKDVCIVPG.KGGGTNALFIKNP.SKYRVKYYGSSFLTHCSIATDSGQDFEIYDSFMAGTDIDEPED
WP_012562171.1 VALISAAERSRVALAPACDDGGTNALACDVV.GRIPLCFGPGSFARHIAAANAADVRPAVLLNQRLGLDLDEPHH
WP_090611448.1 LALLDRPADPDRVVICASADGGTNALLLPLP.ARMDFHYGPGSFMAHCAAARAAGLDPETPALASLRHDIDRQED
WP_011747445.1 LRLLDRPAAPDLAVVCASADGGTNALILPLP.ARMRFRYGQGSFRAHLAEAGAAGLHTLAPALGSLRHDVDRRED
WP_028223137.1 RRLLALSQRYELTLA.ESHDGGTNYLALAPP.QPFIFHYGPQSAALHTRSAQECELSVARCRLPFLARDIDTIDD
WP_041812218.1 GEALAAARTGGRSVVVDHHGTGTAALFSCDPEIPLDPRFGPGSAKRHLE.SGARPLD...GHWPGLRTDVDTADD
WP_033240816.1 ERVLAAAAAHPRSFLADAAGMGTTLLAAAPG.APFTPRFGGPSRARHAA.AGAVELAP..GAVPGVRRDVDTPAD
WP_012642646.1 RHALALLESPGVVLAPDRHGTGTNLLALAPP.DVIAPAFGPFSRWRHRRAAAGARCTIRELWSRALVLDLDTPED
α7 α8
200 210
Mtb-FbiD LTAAR.QLGVGPATARAVAH..R.......................
WP_047154807.1 LLLLK.TMGEGTKTREYLER..YQIFD.KLLTGKVTL...........
WP_010903420.1 LVDVF.VHNTR.RTREWLIAGGWRLAVDDGTPTVVREPND........
WP_011034415.1 LVELL.IHGKGAAKDYIESK..FRLEVKKGRVGLVPL...........
WP_012562171.1 LMQFL.DRGTSTQTDAYLRV..LRLKERKGHSFVVAAQQATGARRLAV
WP_090611448.1 LSALL.THPLRATLAEVRP...........................
WP_011747445.1 LLGFS.LLHHAPMTVEARP...........................
WP_028223137.1 CLVLAHARGRRLPSR...............................
WP_041812218.1 LDAAQ.ALGVGPATRAALDA..LEHTD.PSRCGNE.............
WP_033240816.1 LRAAR.ELGPGPHTSAALAA..LREAA.APRASTAPPG..........
WP_012642646.1 LALVH.RVREGER.................................

Supplementary Figure 1. Conservation of PEP binding residues between *Mtb*-FbiD and *Mj*-CofC.

(a) Superposition of the *Mtb*-FbiD (wheat) on to that of *Mj*-CofC (green) indicates conservation of the residues in the PEP binding site of both proteins. The only change in the binding site, Gly>Tyr in *Mj*-CofC, is not likely to affect binding considering that the hydrogen bond interaction takes place between the backbone nitrogen atom and the oxygen atom of PEP carboxylate group. Protein side chains are shown in ball-and-stick model and labelled with the corresponding color. **(b)** Multiple sequence alignment of FbiD/CofC homologues from *Aneurinibacillus tyrosinisolvens* (WP_047154807.1), *Halobacterium salinarum* (WP_010903420.1), *Methanosarcina mazei* (WP_011034415.1), *Oligotropha carboxidovorans* (WP_012562171.1), *Paracoccus alcaliphilus* (WP_090611448.1), *Paracoccus denitrificans* (WP_011747445.1), *Paraburkholderia oxyphila* (WP_028223137.1), *Rhodococcus jostii* (WP_041812218.1), *Streptomyces griseus* (WP_033240816.1), *Thermomicrobium roseum* (WP_012642646.1). The three -aspartate motif is absolutely conserved.

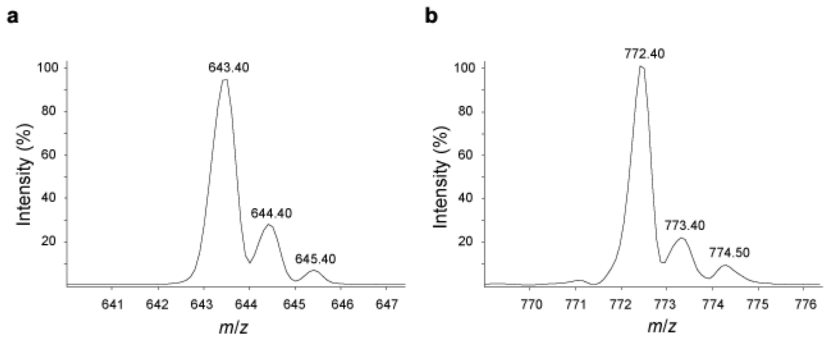

Supplementary Figure 2. Addition of L-glutamate residues to dehydro-F_{420}-0. Dehydro-F_{420}-1 ($[M+H]^+$, monoisotopic *m*/*z* of 643.40, panel (**a**)) and dehydro-F_{420}-2 ($[M+H]^+$, monoisotopic *m*/*z* of 772.40, panel (**b**)) are formed upon the addition of one and two L-glutamate residues, respectively, to dehydro-F_{420}-0.

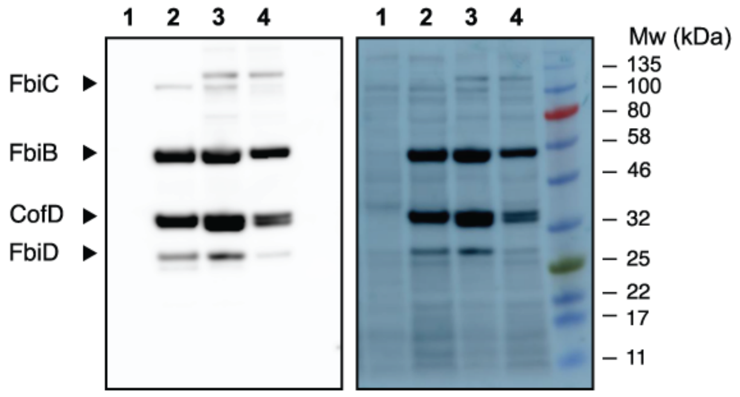

Supplementary Figure 3. Expression of F_{420} biosynthetic genes in *E. coli*. Whole cell lysates were resolved on SDS-PAGE, then immunoblotted with anti-FLAG antibodies to detect expression of F_{420}. 1) Vector-only control 28 °C; 2) pF_{420}-FLAG expressed at 37 °C; 3) pF_{420}-FLAG expressed at 28 °C; pF_{420}-FLAG expressed at 18 °C.

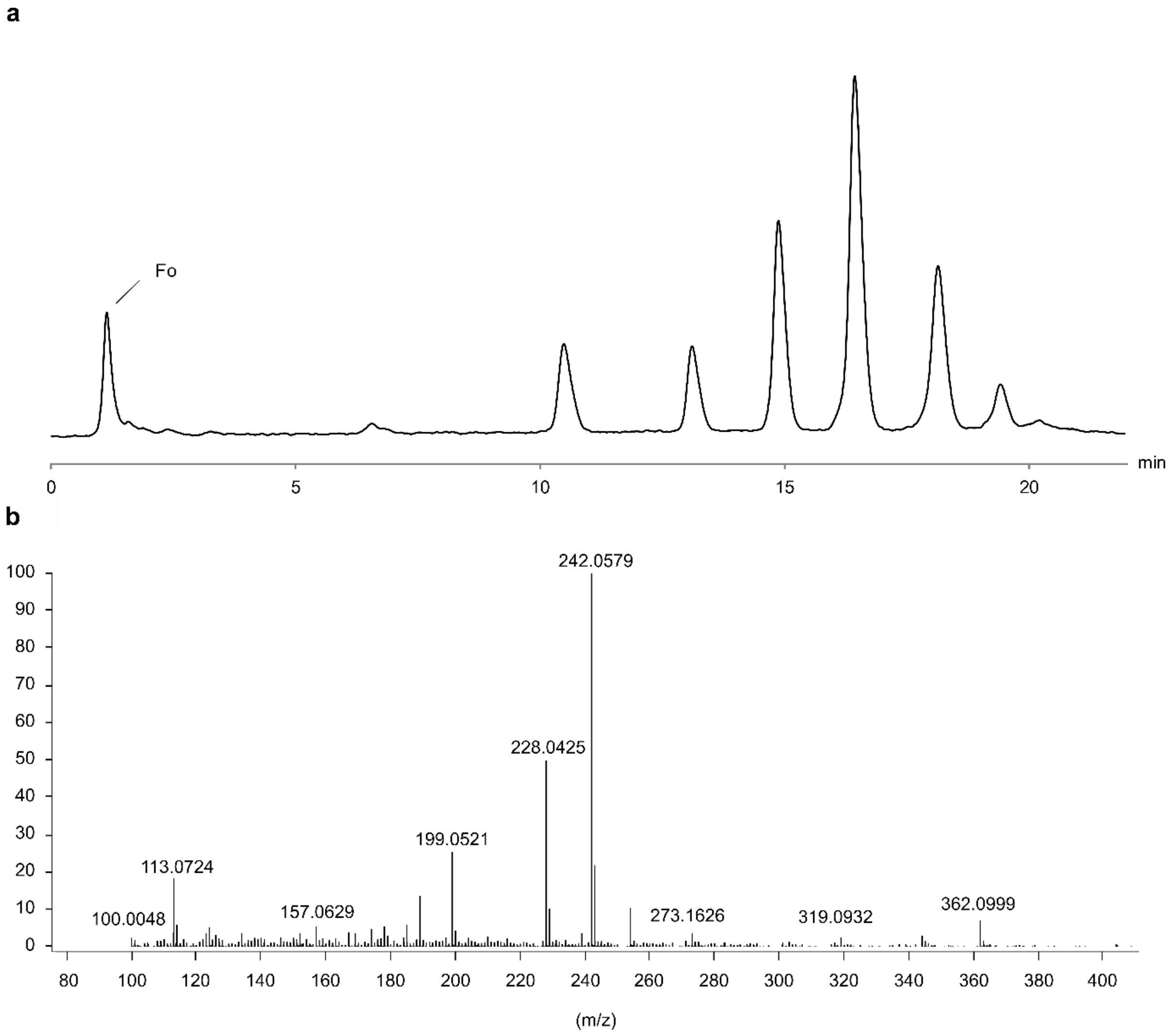

Supplementary Figure 4. Detection of F_{420} biosynthesis intermediate Fo. (a) HPLC-FID trace of *E. coli* lysates containing pF_{420} sampled within 1 hour of induction. **(b)** Fragmentation of Fo obtained from culture media of *E. coli* containing pF_{420}.

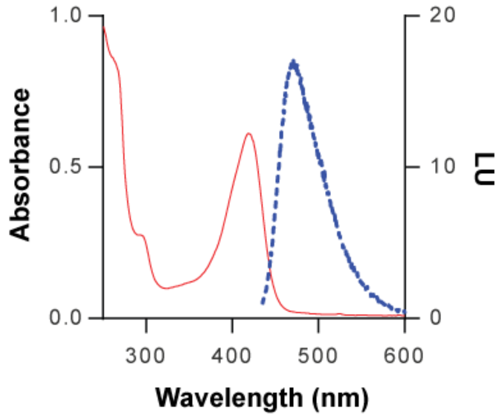

Supplementary Figure 5. Spectrophotometric identification of *E. coli*-produced F_{420}. UV-Vis scan (red) and fluorescence emission (blue) spectra indicate the characteristic features of F_{420}; maximum absorption at 420 nm and maximum fluorescence emission at 480 nm.

Supplementary Table 1. Primers used in the amplification of the constructs used in this study

Construct	Primer Sequences (5′-3′)		Restriction site
pYUBDuet-*fbiD*	Forward	CAGGATGGATCCAGTGAGCGGCACACCGG	BamHI
	Reverse	GTCCCGGAAGCTTCAACGATGTGCGACCGC	HindIII
pYUBDuet-*fbiAB*	Forward	GAAGGAGATATACATATGAAGGTCACCGTTCTG	NdeI
	Reverse	GCCGGCCTTAATTAATCACTTCAGGATCAG	PacI
pYUB28b-*fbiA*	Forward	CAATGACATATGAAAGTTACCGTTCTGGC	NdeI
	Reverse	GCTAGTTATTGCTCAGCG	-
pProEX-*fre*	Forward	ATTAAATAAGGCGCCATGACAACCTTAAGCTG	KasI
	Reverse	TAATAAAAGCTTCAGATAAATGCAAACGC	HindIII
pETMCSIII-*fbiD*	Forward	GTTTAATCGGATCCTAAGGAGGTTAATATTATG	-
	Reverse	GTTAGCAGCCGGATCTATCGATGCATGCCATGGTAC	-

Supplementary Table 2. Kinetic parameters of FGD with F_{420} purified from *M. smegmatis* and *E. coli*

	$k_{cat\ (app)}$ (s^{-1})	$K_{m\ (app)}$ (μM)
M. smegmatis	1.19 ± 0.05	79 ± 11
E. coli	0.46 ± 0.02	77 ± 7

Supplementary Table 3. Malachite green decolorization by recombinant *E. coli* strains

**Strain**	**OD_{615}**
BL21(DE3)	0.199 ± 0.017
BL21(DE3) pFGD_2027 (induced)	0.191 ± 0.017
BL21(DE3) pF_{420} (induced)	0.086 ± 0.002
BL21(DE3) pFGD_2027:pF_{420} (induced)	0.055 ± 0.003
BL21(DE3) pFGD_2027:pF_{420} (uninduced)	0.231 ± 0.012
